# Medical and Demographic Analysis of Health Status of the Population of Some Countries Worldwide

**Published:** 2019-06

**Authors:** Assiya TURGAMBAYEVA, Aigul ISMAILOVA, Gulzhan DOSSYBAYEVA, Gulnaz NUSKABAEVA, Nurlan NAURYZOV, Zauresh MUKHAMETZHANOVA, Karlygash KAIYRBEKOVA, Sona SARGSYAN, Ani MATEVOSYAN, Nargiz ZULKHASH, Aiman MUSINA

**Affiliations:** 1. Department of Public Health №1, Medical University NJSC, Nur-Sultan, Kazakhstan; 2. Department of Ecology, S. Seifullin Kazakh Agro Technical University, Nur-Sultan, Kazakhstan; 3. Department of General Practitioner N2, South Kazakhstan Medical Academy, Chimkent, Kazakhstan; 4. Department of Special Clinical Disciplines, International Kazakh-Turkish University Named after Khoja Akhmet Yassawi, Turkestan, Kazakhstan; 5. Department of Morphology and Physiology, Medical University NJSC, Karaganda, Kazakhstan; 6. Department of ENT Diseases, Medical University Named after M. Heratsi, Yerevan, Armenia; 7. Department of Children’s Policlinic N9, Yerevan, Armenia; 8. Department of Hygiene, Medical University NJSC, Nur-Sultan, Kazakhstan

## Dear Editor-in-Chief

Medical and demographic analysis is one of the most important methods in assessment of the population health status. When characterizing the public health and demographic determinants, such environment of human activities as provision of the person with the right to life and health is also in question.

We aimed to assess the medical and demographic analysis and make a forecast of the main determinants concerning the health status of the population of some countries worldwide. We assessed health status in some countries. The search strategies for other databases were identical. The search was limited to English articles. PRISMA guidelines 2009 for systematic reviews and meta-analyses were reviewed throughout the study.

The maximum peak of the relative growth rate of the world population was recorded in the 60^th^ years of the twentieth century, when it was 2.1% per year. By 2000, the value of this important indicator dropped to 1.33% (1–3).

A list of 24 most populous countries worldwide by the middle of 2015 is depicted in Table 1.

**Table 1:** Most populous countries worldwide (2015)

***№***	***Country***	***Population***	***№***	***Country***	***Population***
1	China	1 374 220 000	13	Vietnam	91 700 000
2	India	1 282 790 000	14	Ethiopia	99 465 819
3	USA	322 613 000	15	Germany	81 292 400
4	Indonesia	258 705 000	16	Egypt	90 197 800
5	Brazil	205 463 000	17	Iran	78 925 800
6	Pakistan	192 457 328	18	Turkey	77 695 904
7	Nigeria	186 988 000	19	Thailand	65 219 369
8	Bangladesh	159 685 000	20	D.R. Congo	85 026 000
9	Russia	146 495 530	21	France	66 539 000
10	Japan	126 880 000	22	United Kingdom	65 572 409
11	Mexico	122 273 500	23	Italy	60 685 487
12	Philippines	102 632 700	24	South Africa	54 956 900

Projections indicate that, being ahead of China in approximately 2025, India will become the most populous country in the world in 2050. The USA will occupy the third place - with a population of 423 million people (308 million people in 2010). Fall in birth rate of two countries, Japan and Russia, from among the economically and politically influential countries likely will lead to the fact that they will lose their current position, having moved from the 9th and 10th place to the 16th and 17th places respectively. These are the results of population size assessment and projections for 228 countries compiled by the US Census Bureau.

The publication highlights such predictions: by 2050, the rapid growth of China’s population will slow down, and population growth in Western Europe will increase, particularly in Spain and Italy, both due to birth and due to immigration (2, 4, 5).

**Fig. 1:**
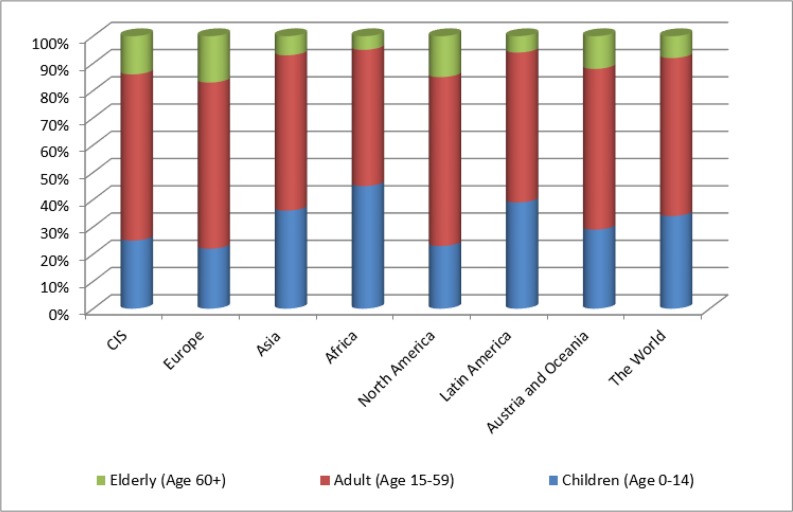
Percentage of different aged population groups in the total population, %

Fig. 1 shows the division of the world’s population into three age groups in 2015. In developed countries, the proportion of children in the total population is an average of 23% and 15% of elderly people, 43% and 6% respectively - in developing countries.

The data in Table 2 shows how the average life expectancy (ALE) was increased in some regions of the Earth since 2000 till 2015.

Thus, by 2030, the world’s ageing process will be expressed quite clearly. An attention of the government agencies should be focused exactly on the problem of the “third” (golden) age people to reduce the negative impact of socio-economic factors that affect the population health.

**Table 2:** Dynamics of average life expectancy (ALE) in certain regions of the earth in 2000–2015

***Region ***	***Africa***	***America***	***Southeast Asia***	***Europe***	***The Mediterranean***	***The World***
***Year ***						
2000	50.6	73.7	63.5	72.3	65.4	66.4
2001	51.0	74.0	63.9	72.5	65.5	66.7
2002	51.3	74.2	64.3	72.6	65.8	67.0
2003	51.7	74.4	64.7	72.7	65.7	67.2
2004	52.2	74.7	64.7	73.2	66.1	67.6
2005	53.0	74.9	65.4	73.2	66.1	68.0
2006	53.8	75.3	65.7	73.9	66.5	68.4
2007	54.6	75.3	66.1	74.3	66.8	68.8
2008	55.5	75.6	66.3	74.6	67.2	69.1
2009	56.3	75.9	66.8	75.0	67.5	69.6
2010	57.0	75.3	67.2	75.3	67.9	69.8
2011	57.6	76.2	67.6	75.8	68.2	70.3
2012	58.2	76.4	68.0	76.0	68.1	70.5
2013	58.8	76.5	68.3	76.3	68.4	70.8
2014	59.3	76.7	68.7	76.5	68.5	71.1
2015	60.0	76.9	69.0	76.8	68.8	71.4
